# The Treatment of Fractures by Mobilisation and Massage

**Published:** 1912-03

**Authors:** 


**The Treatment of Fractures by Mobilisation and Massage.** By JAMES B. MENNELL, M.D., B.C. Pp. xvi, 458. London: Macmillan & Co. Limited. 1911.

This book is written by a late House Surgeon of St. Thomas's Hospital who has been in charge of the Casualty and Physical Exercise Departments. It sets out the author's personal experiences in the treatment of fractures by the methods advocated by Professor Lucas-Championnière, who writes a Preface in French for it. These experiences are based upon upwards of 400 cases, almost all of which have been out-patients or casualties. It follows from this that cases of such gravity as required their admission as in-patients have not come under the author's observation. The sections dealing with fractures of the lower extremity are therefore of very much less value than the rest of the book.

As an exposition of the Mobilisation and Massage method in the treatment of fractures it is most clear and convincing, and to English readers it is of particular value because it gives an understanding of Professor Lucas-Championnière's methods, which it is difficult to obtain from any other English source. For example, the term "massage" conveys quite a false representation to the English reader of this method of treating fractures, unless it is very carefully qualified and explained.

This is most clearly shown by the statement that this treatment ought never to be trusted to a professional masseur. The cardinal principle underlying the whole method is the relief of pain and muscular spasm by means of stroking so light as to be compared to stroking a cat or mesmeric passes. The rationale of this principle is discussed and explained in the present volume with the utmost clearness. This light stroking massage, if successfully carried out, appears to act with greater potency than the most profound anæsthesia, for it not only abolishes pain, relaxes the muscles, and allows a re-position of the fragments, but it maintains these desirable conditions for a considerable length of time.

So soothing is the treatment, that the patient often falls to sleep while it is being carried out. So remarkable indeed is the resemblance of this method to the phenomena of hypnosis, that one is tempted to ask the question whether the two processes are not one and the same. Is the success of this treatment dependent upon the possession of a special occult gift on the part of the operator?

Dr. Mennell's criticisms of other methods of treating fractures are naturally somewhat extreme, but perhaps they are fair and reasonable. The classical method of long immobilisation on a splint is everything that is evil; operative treatment for fractures is to be reserved for those cases in which light stroking fails to effect a reduction; the method of continuous weight extension, advocated chiefly by Bardenheuer, is scarcely mentioned. None of the advocates of extreme views relating to the treatment of fractures pay sufficient attention to the discussion of the special kind of cases suitable for their method. The vast bulk of medical practitioners in England and the continent are accustomed to obtain good results from the splint method of treating fractures in most of their cases. The method advocated in the present volume, although it does not involve any risk to the patient's life, is contrary to the prejudices of the public, and it demands an excessive amount of personal attention on the part of the medical man. We think, therefore, it will never be generally adopted until its authors can more precisely indicate the special type of case in which it is demanded. The book is throughout well written and illustrated, and it forms a most welcome and valuable addition to the English literature on this subject.

